# A Full Pharmacological Analysis of the Three Turkey β-Adrenoceptors and Comparison with the Human β-Adrenoceptors

**DOI:** 10.1371/journal.pone.0015487

**Published:** 2010-11-30

**Authors:** Jillian G. Baker

**Affiliations:** Wellcome Trust Clinician Scientist Fellow, Institute of Cell Signalling, University of Nottingham, Nottingham, United Kingdom; University of South Florida College of Medicine, United States of America

## Abstract

**Background:**

There are three turkey β-adrenoceptors: the original turkey β-adrenoceptor from erythrocytes (tβtrunc, for which the X-ray crystal structure has recently been determined), tβ3C and tβ4C-receptors. This study examined the similarities and differences between these avian receptors and mammalian receptors with regards to binding characteristics and functional high and low affinity agonist conformations.

**Methodology/Principal Findings:**

Stable cell lines were constructed with each of the turkey β-adrenoceptors and ^3^H-CGP12177 whole cell binding, CRE-SPAP production and ^3^H-cAMP accumulation assays performed. It was confirmed that the three turkey β-adrenoceptors are distinct from each other in terms of amino acid sequence and binding characteristics. The greatest similarity of any of the turkey β-adrenoceptors to human β-adrenoceptors is between the turkey β3C-receptor and the human β2-adrenoceptor. There are pharmacologically distinct differences between the binding of ligands for the tβtrunc and tβ4C and the human β-adrenoceptors (e.g. with CGP20712A and ICI118551). The tβtrunc and tβ4C-adrenoceptors appear to exist in at least two different agonist conformations in a similar manner to that seen at both the human and rat β1-adrenoceptor and human β3-adrenoceptors. The tβ3C-receptor, similar to the human β2-adrenoceptor, does not, at least so far, appear to exist in more than one agonist conformation.

**Conclusions/Significance:**

There are several similarities, but also several important differences, between the recently crystallised turkey β-adrenoceptor and the human β-adrenoceptors. These findings are important for those the field of drug discovery using the recently structural information from crystallised receptors to aid drug design. Furthermore, comparison of the amino-acid sequence for the turkey and human adrenoceptors may therefore shed more light on the residues involved in the existence of the secondary β-adrenoceptor conformation.

## Introduction

There are three human β-adrenoceptors, β1, β2 and β3 and all are Gs-coupled GPCRs. Following observations by Pak and Fishman [1], recent studies from several groups (including this laboratory) have suggested that the human β1-adrenoceptor [2]–[13] and β3-adrenoceptor [14], so far in contrast to the β2-adrenoceptor, exist in two agonist states or conformations. The high affinity “catecholamine” conformation is where catecholamines and many other agonists act and these agonist responses are readily inhibited by β-blockers. Agonist responses are also seen via stimulation of the secondary or lower affinity conformation but these agonist responses require significantly higher concentrations of antagonist to be inhibited [2]–[14]. Ligands have also been found to have different efficacies at the two conformations with some ligands being agonists of both conformations (e.g. alprenolol, pindolol; [10], including work from this laboratory [11]), others acting as antagonists at both sites (albeit with markedly different affinities e.g. CGP20712A, bisoprolol; [6]–[7], including previous studies from this laboratory [9]) and others acting as antagonists at the high affinity site whilst stimulating an agonist response via the lower affinity site (i.e. at higher concentration of ligand e.g. CGP12177 carvedilol; [6] [11]–[12]. The precise nature of this second conformation however remains uncertain. It appears that, at least for the human β1-adrenoceptor, some of the same residues are required by both conformations of the receptor and therefore the binding site for the classical catecholamine site and the secondary site must overlap to some degree [13].

The turkey has a β-adrenoceptor expressed on erythocytes that has been studied for many years [15]–[18]. However Nicholas and colleagues identified two further turkey β-adrenoceptors, the turkey β3C and turkey β4C-adrenoceptors [19]. They conclude that the turkey β4C-adrenoceptor is distinct both in amino-acid sequence and pharmacological characteristics from the original turkey β-adrenoceptor. They also mention a turkey β3C-adrenoceptor that appears similar to the mammalian β2-adrenoceptor, although its sequence and pharmacological characterisation are not presented. It is not known whether these avian receptors exist in one or more agonist conformations or how pharmacologically similar they are, or not, to the human β-adrenoceptors. Knowledge of more active agonist conformations may assist in determining which other residues in the human receptors are necessary for this property.

The X-ray crystal structure has recently been determined for the human β2-adrenoceptor and the original turkey β-adrenoceptor [20]–[23]. This is potentially of huge importance for those in the field of drug discovery who are using computational models of these receptors to assist in the process of ligand design. An important question is how similar, if at all, is the pharmacology of the original turkey β-adrenoceptors to any of the human β-adrenoceptors.

Thus there are two important reasons to understand the detailed pharmacological properties of the three turkey β-adrenoceptors: 1) to understand the similarities and differences between the human and the original turkey β-adrenoceptor (where structural information in now available) to understand how best to use this information in the field of drug design and 2) to investigate whether any of these have more than one active agonist conformation and so understand more about the residues involved that give some β-adrenoceptors the high and low affinity states.

This study therefore examines in detail the pharmacology of the three turkey β-adrenoceptors, firstly to determine their pharmacological similarity to each other, then the similarity to the three human β-adrenoceptors and finally to determine whether or not they exist in more than one active agonist conformation.

## Methods

### Materials

Foetal calf serum which was from PAA Laboratories (Teddington, Middlesex, UK). Microscint 20 scintillation fluid from PerkinElmer (Shelton, CT, USA). ^3^H-CGP12177, ^3^H-adenine and ^14^C-cAMP were from Amersham International (Buckinghamshire, UK). Betaxolol, bisoprolol, bucindolol, BRL37344, carvedilol, cimaterol, formoterol, ICI215001, isoprenaline, L755507, salmeterol, SDZ21009, SR59230A, ZD2079 and ZD7114 were from Tocris Life Sciences (Avonmouth, UK). Carazolol was a gift from Dr Christopher Tate (LMB, Cambridge, UK); nebivolol and butoxamine were a gift from Stefano Evangelista (Menarini Ricerche Spa, Florence, Italy); zinterol was a gift from Dr Torsten Christ (Department of Pharmacology and Toxicology, Dresden University of Technology, Germany.). All other reagents were from Sigma Chemicals (Poole, Dorset, UK).

### Cell lines and cell culture

CHO-K1 stably expressing a CRE-SPAP (secreted placental alkaline phosphatase) reporter gene [24] were secondarily transfected with either the original full length turkey β-adrenoceptor, the turkey β3C-adrenoeptor or the turkey β4C-adrenoceptor (all gifts from Dr Rob Nicholas, University of North Carolina, USA; all on geneticin selection marker) using Lipofectamine and OPTIMEM as per manufacturer's instructions. Despite multiple efforts the original full length turkey β-adrenoceptor did not express. A C-terminally truncated version was therefore transfected (tβtrunc, gift from Dr Chris Tate, LMB, Cambridge, UK). This mutant has 59 amino-acids deleted from the C-terminus and one point mutation C116L [23]. For transient transfections (initial binding experiments only, see Results), the parent CHO-SPAP cells were transfected on day 1, the transfection reagents removed and replaced with media on day 2, the cells plated in to white-sided 96 well plates on day 3 and a binding assay performed on day 4. To generate stable cells lines (as used for all the binding and functional data presented in this manuscript), cells were selected for 3 weeks using resistance to geneticin (1 mg/ml for the receptor) and hygromycin (200 µg/ml for the CRE-SPAP reporter). Single clones were then isolated by dilution cloning. Cells were grown in Dulbecco's modified Eagle's medium nutrient mix F12 (DMEM/F12) containing 10% foetal calf serum and 2 mM L-glutamine in a 37°C humidified 5% CO_2_: 95% air atmosphere.

### ^3^H-CGP12177 Whole Cell Binding

Cells were grown to confluence in white-sided tissue culture treated 96-well view plates. ^3^H-CGP12177 whole cell binding was performed as previously described [25]. For saturation binding, the media was removed from each well, then 100 µl serum-free media (DMEM/F12 containing 2 nM L-glutamine) or 100 µl propranolol 20 µM added to each well. ^3^H-CGP 12177 (in 100 µl, thus a 1∶2 dilution in the well, to give final well concentrations of 0.02 nM to 25.84 nM for ^3^H-CGP12177 and 10 µM propranolol to define non-specific binding) was added immediately and the plates incubated for 2 hours at 37°C, 5% CO_2_, humidified atmosphere. For competition binding, the media was removed from each well, 100 µl of serum-free media containing the competing ligand at twice the final required concentration was added to each well followed immediately by a 100 µl of a fixed concentration of ^3^H-CGP12177 (1∶2 dilution in well, final well concentrations of 1.24 nM to 12.10 nM). The cells were incubated for 2 hours at 37°C, 5% CO_2_ humidified atmosphere. For all plates, the cells were washed twice by the addition and removal of 200 µl 4°C phosphate buffered saline. 100 µl Microscint 20 was added to each well, a white sticky base applied to the underside and a sealant top applied to the top of the plate. The plates were left at room temperature overnight in the dark and the plates counted on a Topcount at 21°C for 2 minutes per well.

### CRE-SPAP production

Cells were grown to confluence in clear plastic tissue culture treated 96-well plates. The cells were then serum starved for 24 hours before experimentation by removal of the media and addition of 100 µl serum free media per well. At the start of each experiment, 100 µl serum free media or 100 µl serum free media containing an antagonist at the final required concentration was added to the wells and the cells incubated for 30 minutes at 37°C. Agonist in 10 µl was then added to each well and the plates incubated for 5 hours. After 5 hours, the media and all drug were removed from each well and 40 µl serum free media added to each well. The plates were then incubated for 1 hour at 37°C. The plates were then transferred to an oven preheated to 65°C and incubated for 30 minutes to destroy endogenous phosphatases. SPAP production was then measured by the addition of 100 µl 5 mM p-NPP per well (in diethanolamine buffer) and read on a Dynatech MRX plate reader at 405 nM.

### ^3^H-cAMP accumulation

Cells were grown to confluence in clear plastic tissue culture treated 24-well plates. The media was removed and the cells pre-labelled with ^3^H-adenine by incubation for 2 hours with 2 µCi/ml ^3^H-adenine in serum-free media (0.5 ml per well). The ^3^H-adenine was removed and each well washed by the addition and removal of 1 ml serum-free media. 1 ml serum-free media containing 1 mM IBMX was added to each well and the cells incubated for 15 minutes. Agonists (in 10 µl serum-free media) were added to each well and the plates incubated for 5 hours. Where CGP 12177 and cimaterol were co-incubated with the cells, the two ligands were added together after the 15 minute IBMX incubation. The reaction was terminated by the addition of 50 µl concentrated HCl per well. The plates were then frozen, thawed and ^3^H-cAMP separated from other ^3^H-nucleotides by sequential Dowex and alumina column chromatography, as previously described [26].

### Data analysis

#### Whole cell binding - Saturation binding

Saturation binding curves of the total and non-specific binding (as determined by the presence of 10 µM propranolol) were performed in order to determine the specific binding K_D_ value for ^3^H-CGP12177 and the receptor expression level. All data points on each binding curve were performed in quadruplicate. Specific binding (SB, equation 1) of ^3^H-CGP12177 at different concentrations of the ^3^H-ligand was fitted using the non-linear regression program Prism 2.01 to the equation: 
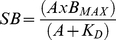
(1)where A is the concentration of ^3^H-CGP12177, B_MAX_ is the maximal specific binding and K_D_ is the dissociation constant of ^3^H-CGP12177. Protein was determined by using a Pierce BCA Protein Assay Kit (Thermo Scientific, Rockford, USA) as per manufacturers' instructions.

#### Whole cell binding - Competition binding

All data points on each binding curve were performed in triplicate and each 96-well plate also contained 3–6 determinations of total and non-specific binding. In all cases where a K_D_ value is stated, the competing ligand completely inhibited the specific binding of ^3^H-CGP12177.

A one-site sigmoidal response curve was then fitted to the data using Graphpad Prism 2.01 and the IC_50_ was then determined as the concentration required to inhibit 50% of the specific binding.
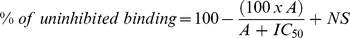
(2)where A in the concentration of the competing ligand, IC_50_ is the concentration at which half of the specific binding of ^3^H-CGP12177 has been inhibited, and NS is the non-specific binding.

From the IC_50_ value and the known concentration of radioligand [^3^H-CGP12177], a K_D_ (concentration at which half the receptors are bound by the competing ligand) value was calculated using the equation: 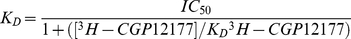
(3)


#### Functional assays - 3H-cAMP accumulation and CRE-SPAP production

Most agonist responses were best described by a one-site sigmoidal concentration response curve (equation 4)
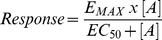
(4)


Where Emax is the maximum response, [A] is the agonist concentration and EC50 is the concentration of agonist that produces 50% of the maximal response

The affinities of antagonists were calculated (K_D_ values) from the shift of the agonist concentration responses in the presence of a fixed concentration of antagonist using equation 5:
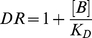
(5)where DR (dose ratio) is the ratio of the agonist concentration required to stimulate an identical response in the presence and absence of a fixed concentration of antagonist [B].

In experiments where 3 different fixed concentrations of the same antagonist were used, Schild plots were constructed using the following equation 6:
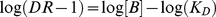
(6)


These points were then fitted to a straight line. A slope of 1 then indicates competitive antagonism [27].

When CGP12177 was used to antagonise the more efficacious agonists, clear partial agonism was seen. Here, the affinity was initially calculated by the method of Stephenson [28] using equation 7:
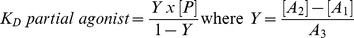
(7)where [P] in the concentration of CGP12177, [A_1_] in the concentration of the agonist at the point where CGP 12177 alone agonist causes the same response, [A_2_] is the concentration of agonist causing a given response above that achieved by the partial agonist and [A_3_] the concentration of the agonist, in the presence CGP12177, causing the same stimulation as [A_2_].

Several of the responses were however best fitted to a two-site concentration response – equation 8 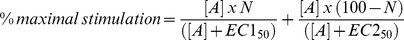
(8)where N is the percentage of site 1, [A] is the concentration of agonist and EC1_50_ and EC2_50_ are the respective EC_50_ values for the two agonist sites.

A two-site analysis was also used for some experiments equation 9:
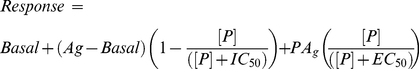
(9)where basal is the response in the absence of agonist, Ag is the response to a fixed concentration of agonist, [P] is the concentration of partial agonist (e.g. CGP12177), IC_50_ is the concentration of competing partial agonist that inhibits 50% of the response of the fixed agonist, PAg is the maximum stimulation by the competing partial agonist and EC_50_ is the concentration of competing agonist that stimulated a half maximal competing partial agonist response.

A 10 µM (maximal) isoprenaline concentration was included in each plate for each separate experiment for CRE-SPAP production and ^3^H-cAMP accumulation, to allow agonist responses to be expressed as a percentage of the isoprenaline maximum for each experiment. All data are presented as mean ± s.e.m. of triplicate determinations (except saturation binding experiments where determinations were from quadruplicate wells) and n in the text refers to the number of separate experiments.

## Results

**Figure 1:**
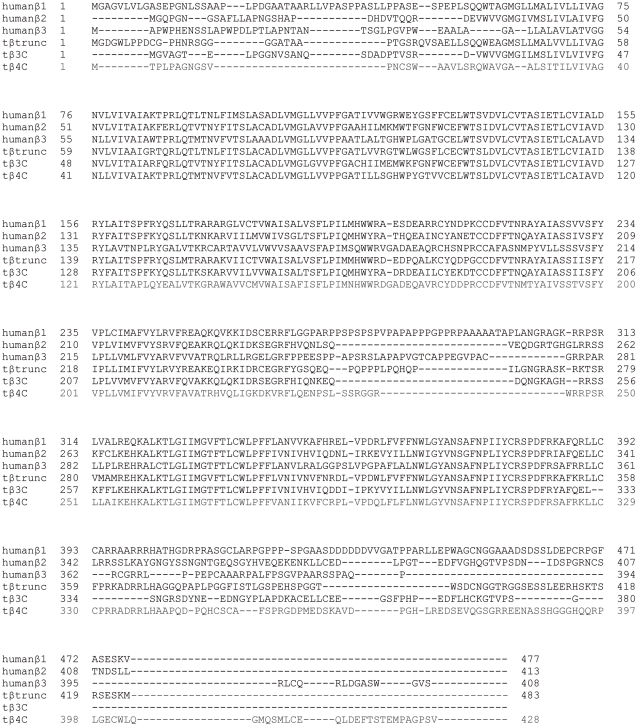
The amino acid sequence of the three turkey and the three human β-adrenoceptors. Amino acid sequences shown for published receptors are taken from UniProtKB/Swiss-Prot and correspond to the translation of the coding sequence from each nucleotide receptor construct used in the present study. All receptor constructs were checked by sequencing on both strands using automated fluorescent sequencing (University of Nottingham, Nottingham, U.K.).

The amino acid sequence of the three turkey β-adrenoceptors and the three human β-adrenoceptors is given in Figure 1.

### ^3^H-CGP12177 whole cell binding

Binding studies were performed on both transiently transfected cells and the stable cell lines to ensure that the stable cell line was indeed a true reflection of the pharmacology of the transfected β-adrenoceptor and that no alteration of the receptor had taken place during the process of making the cell line. The results from 8 separate transiently transfected cell populations (both K_D_ of ^3^H-CGP12177 from saturation studies and K_D_ values of betaxolol, bisoprolol, clenbuterol, CGP20712A, formoterol, ICI118551, metoprolol and xamoterol from competition studies) were very similar to the results obtained from the stable cell line so only the stable cell line data are presented here.

**Figure 2:**
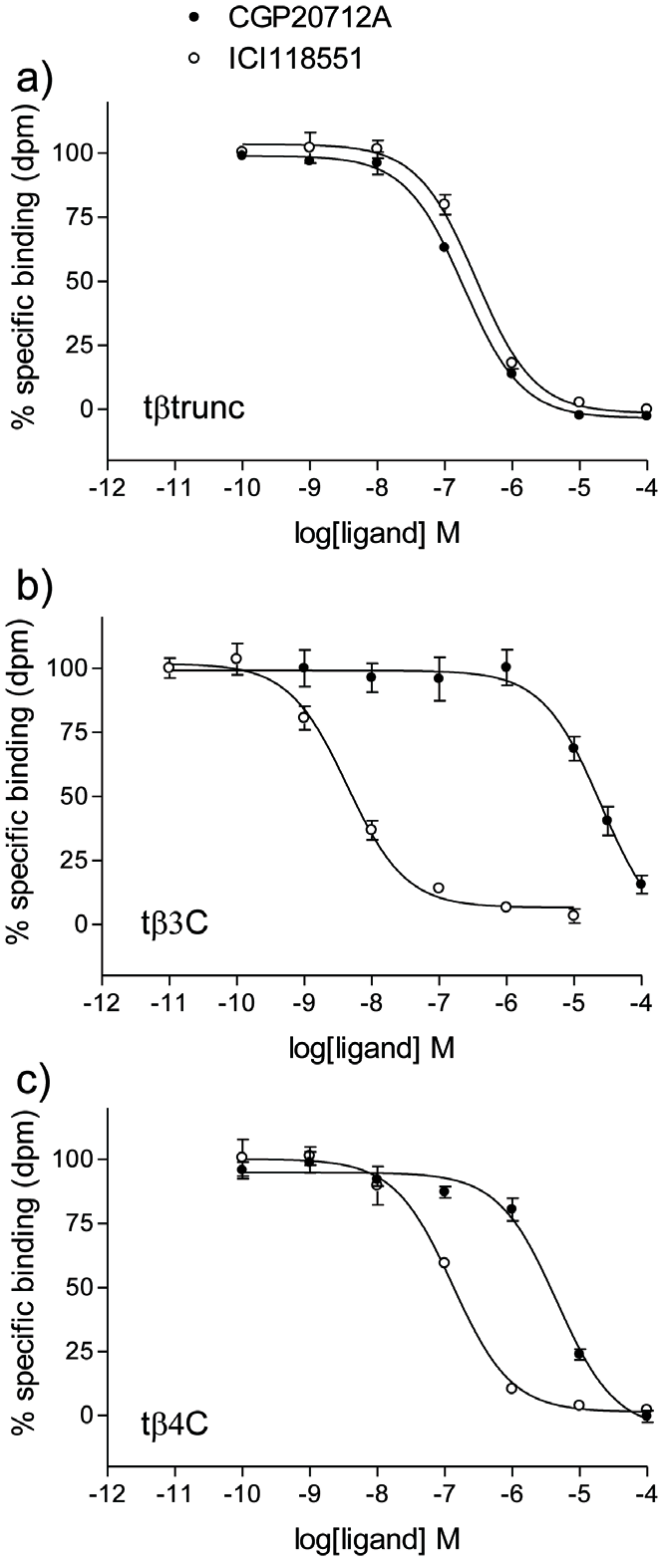
Inhibition of ^3^H-CGP12177 specific binding to whole cells expressing the 3 turkey β-adrenoceptors. Inhibition of ^3^H-CGP12177 specific binding to whole cells by CGP20712A and ICI118551 in a) CHO tβtrunc cells, b) CHO tβ3C cells and c) CHO tβ4C cells. Non-specific binding was determined by 10 µM propranolol. The concentrations of ^3^H-CGP 12177 present in each case are a) 1.24 nM, b) 0.855 nM and c) 1.59 nM. Data points are mean ± s.e.mean of triplicate determinations. These single experiments are representative of a) 5, b) 9 and c) 9 separate experiments.

^3^H-CGP12177 bound specifically to all three turkey β-adrenoceptors to give K_D_ values of 0.42±0.03 nM (n = 12) for the tβtrunc (receptor expression level of 148fmol/mg protein), 0.29±0.02 nM (n = 7) for the tβ3C (63fmol/mg protein) and 0.58±0.06 nM (n = 7) for the tβ4C-receptor (67fmol/mg protein) in the stable cell lines generated. The affinity of a range of other ligands (including agonists, partial agonists and inverse agonists) was then assessed. CGP20712A is a highly selective human β1-antagonist (log K_D_ at human β1 = −8.81; at human β2 = −6.11 [25]) and ICI118551 is a highly selective human β2-antagonist (log K_D_ at human β1 = −6.52; at human β2 = −9.26 [25]). However, these ligands bind with almost equal affinity to the tβtrunc-receptor (−7.53 for CGP20712A and −7.20 for ICI118551; Figure 2, Table 1). ICI118551 however has high affinity, and CGP20712A low affinity, for the tβ3C-receptor in a similar pattern to that of the human β2-adrenoceptor.

**Table 1:** Log K_D_ values obtained from ^3^H-CGP 12177 whole cell binding studies at the three turkey β-adrenoceptors.

	tβtrunc		tβ3C		tβ4C		Human β1	Human β2	Human β3
Acebutolol	−5.85±0.02	6	−5.39±0.09	10	−5.41±0.08	6	−6.46	−6.08	−4.41
Adrenaline	−6.01±0.04	6	−5.81±0.06	9	−5.77±0.08	7	−5.15	−6.13	−4.70
Alprenolol	−7.96±0.04	5	−8.44±0.10	7	−7.85±0.10	7	−7.83	−9.04	−6.93
Atenolol	−5.40±0.06	6	−4.75±0.10	10	−4.76±0.05	7	−6.66	−5.99	−4.11
Betaxalol	−6.90±0.04	6	−6.29±0.08	8	−6.24±0.06	7	−8.21	−7.38	−5.97
BRL35135A	−6.32±0.07	5	−7.42±0.06	5	−6.39±0.03	5	−6.08	−7.38	−6.73
BRL37344	−5.19±0.03	6	−6.28±0.06	7	−5.89±0.10	7	−5.19	−6.51	−6.45
Bisoprolol	−6.70±0.03	6	−5.58±0.08	6	−6.26±0.08	7	−7.83	−6.70	−5.67
Bupranolol	−8.43±0.04	6	−9.07±0.07	7	−8.18±0.09	7	−8.51	−9.85	−7.04
Bucindolol	−9.43±0.06	7	−9.72±0.09	9	−9.41±0.07	6	−9.31	−9.99	−8.04
Butoxamine	−5.24±0.03	6	−5.99±0.08	6	−4.90±0.05	4	−4.85	−6.23	>−4
Carazolol	−10.20±0.05	6	−10.92±0.11	6	−10.27±0.09	6	−9.69	−10.49	−8.35
Carvedilol	−9.43±0.05	5	−9.58±0.07	7	−9.91±0.02	7	−8.75	−9.40	−8.30
CGP20712A	−7.53±0.04	8	−5.40±0.11	9	−6.05±0.07	12	−8.81	−6.11	−5.19
Cimaterol	−7.17±0.03	9	−7.06±0.08	8	−6.75±0.04	8	−6.57	−7.26	−5.28
CL316243	>−4	6	>−4	4	>−4	4	>−3	−4.10	−5.11
Clenbuterol	−6.74±0.07	6	−7.38±0.10	7	−6.44±0.03	7	−6.62	−7.90	−5.35
S-Cyanopindolol	−10.89±0.06	6	−11.37±0.07	6	−10.81±0.09	6	−10.39	−11.09	−8.36
Denopamine	−6.29±0.05	7	−5.63±0.06	6	−5.95±0.03	6	−6.12	−5.83	−5.30
Dobutamine	−5.69±0.03	6	−5.57±0.06	8	−6.00±0.06	7	−5.23	−5.84	−5.09
Fenoterol	−6.36±0.03	6	−6.69±0.07	10	−6.22±0.10	10	−5.04	−7.03	−5.39
Formoterol	−7.33±0.06	6	−7.96±0.07	7	−7.70±0.07	7	−6.11	−8.63	−5.82
ICI118551	−7.20±0.03	5	−8.93±0.06	9	−7.37±0.03	9	−6.52	−9.26	−6.44
ICI215001	−5.79±0.05	6	−5.31±0.09	7	−6.55±0.06	10	−6.37	−5.86	−6.63
Isoprenaline	−6.86±0.08	7	−6.50±0.08	7	−6.71±0.09	7	−6.06	−6.64	−5.52
Labetolol	−7.51±0.05	6	−7.42±0.04	7	−7.70±0.04	6	−7.63	−8.03	−6.18
L755507	−7.17±0.03	7	−6.56±0.06	5	−7.63±0.11	5	−6.23	−6.83	−8.60*
Metaproterenol	−5.64±0.07	6	−5.38±0.09	5	−5.13±0.09	6	−4.71	−5.30	−4.06
Metoprolol	−6.42±0.03	7	−5.96±0.09	6	−5.78±0.07	7	−7.26	−6.89	−5.16
Nadolol	−7.99±0.04	6	−7.95±0.09	6	−7.74±0.03	6	−7.23	−8.60	−6.18
Noradrenaline	−6.60±0.03	6	−4.94±0.07	7	−6.30±0.08	7	−5.74	−5.41	−5.53
Nebivolol	−8.25±0.05	6	−7.78±0.09	8	−8.83±0.08	9	−9.06	−7.92	−7.04
Pindolol	−8.55±0.03	6	−8.71±0.10	8	−8.28±0.06	7	−8.57	−9.23	−7.08
Pronethalol	−6.85±0.01	6	−6.99±0.07	7	−7.06±0.04	7	−6.44	−7.36	−5.89
Propranolol	−8.51±0.05	7	−8.49±0.06	7	−8.51±0.06	7	−8.16	−9.08	−6.93
Ractopamine	−6.75±0.03	6	−6.65±0.04	6	−6.57±0.04	6	−6.97	−6.93	−5.82
Salbutamol	−4.99±0.03	6	−6.14±0.07	7	−5.36±0.05	7	−4.66	−6.12	−4.33
Salmeterol	−5.92±0.04	8	−8.50±0.07	8	−7.09±0.04	8	−5.73	−9.26	−6.33
SDZ21009	−9.27±0.08	6	−10.17±0.08	10	−9.06±0.07	7	−8.94	−10.28	−7.10
SR59230A	−7.39±0.04	7	−7.96±0.08	6	−7.65±0.06	7	−7.54	−8.45	−7.37
Sotalol	−6.14±0.06	6	−6.27±0.08	6	−6.16±0.05	7	−5.77	−6.85	−5.05
Terbutaline	−4.61±0.04	6	−5.62±0.09	10	−4.47±0.05	5	−3.82	−5.62	−3.90
Timolol	−8.76±0.02	5	−9.24±0.10	11	−8.70±0.06	7	−8.27	−9.68	−6.80
Tulobuterol	−5.60±0.06	7	−6.64±0.08	9	−5.46±0.04	7	−5.62	−6.83	−4.72
Xamoterol	−6.58±0.02	6	−5.64±0.09	8	−6.23±0.05	7	−7.22	−6.07	−4.45
ZD2079	−3.77±0.03	4	>−4	4	>−4	4	−4.21	>−4	−4.59
ZD7114	−7.30±0.09	6	−6.59±0.10	7	−7.59±0.04	7	−7.58	−7.31	−6.78
zinterol	−6.40±0.03	6	−7.62±0.08	6	−6.80±0.03	6	−5.96	−8.04	−6.27

Log K_D_ values obtained from ^3^H-CGP 12177 whole cell binding studies in CHO cells stably expressing either the tβtrunc, tβ3C or tβ4C adrenoceptors. The human β1 β2 and β3-data is taken from [25] and [30] and is given for comparison. Values represent mean ± s.e.m. for n separate experiments.

*binding to site 1 [30].

**Figure 3:**
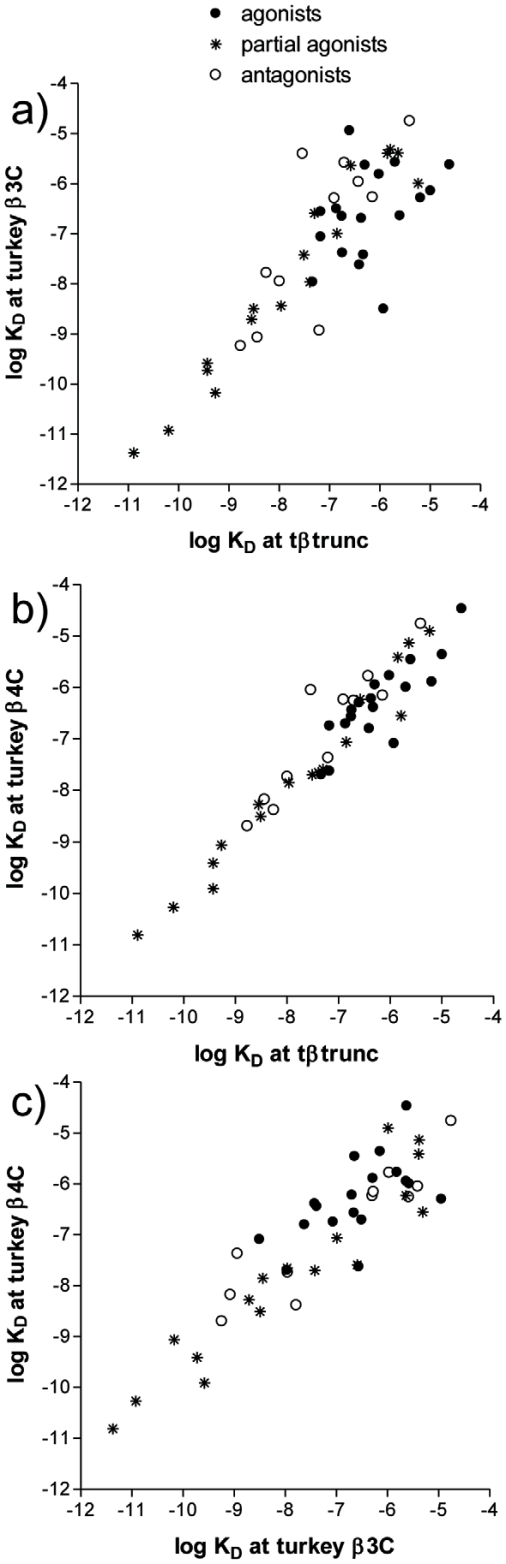
Correlation plot of the log K_D_ values for the turkey β-adrenoceptors compared with each other. Ligands are labelled as full agonists if the stimulated more than 90% of the response at the human β1-adrenoceptor, as partial agonists if they stimulated 5–90% of the full response and as antagonists if they stimulated less than 5% of a full agonist response (data from human β1 taken from [9] [30] [39].

When many ligands are examined it is possible to build up a pattern of affinities and thus judge the similarity or differences between the receptors (Table 1, Figure 3). For comparative purposes, the equivalent binding affinity plot comparisons between the human β-adrenoceptors are given in the Figure S1.

**Figure 4:**
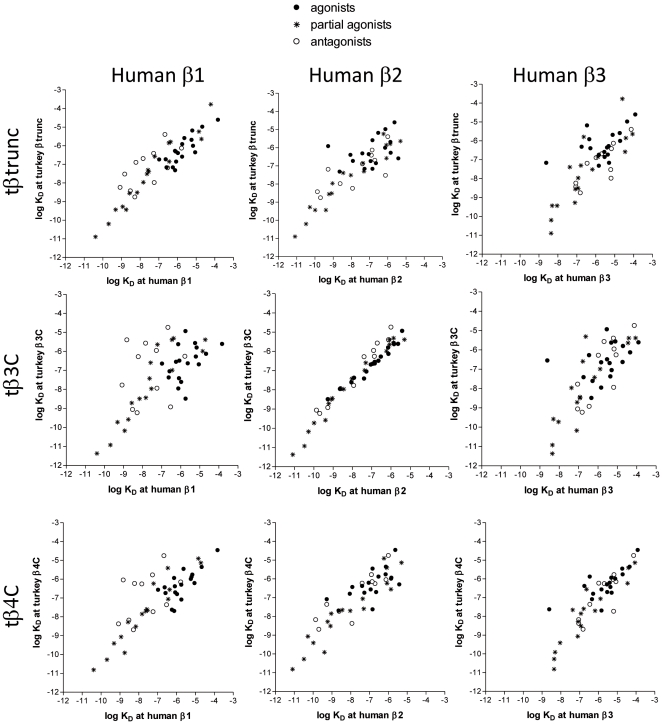
Correlation plot of the log K_D_ values for the turkey β-adrenoceptors compared with the human β-adrenoceptors. Correlation plot of the log K_D_ values for the ligands in Table 1 for the three turkey β-adrenoceptors vs the three human β-adrenoceptors. The human adrenoceptor data is taken from [25] and [30]. Ligands are labelled as full agonists if the stimulated more than 90% of the response at the human β1-adrenoceptor, as partial agonists if they stimulated 5–90% of the full response and as antagonists if they stimulated less than 5% of a full agonist response (data from human β1 taken from [9] [30] [39].

Similarly, the turkey β-adrenoceptors can be compared to the human β-adrenoceptors (Table 1, Figure 4). From this is can be seen that the greatest similarity is between the tβ3C-receptor and the human β2-adrenoceptor.

### CRE-SPAP gene transcription

**Figure 5:**
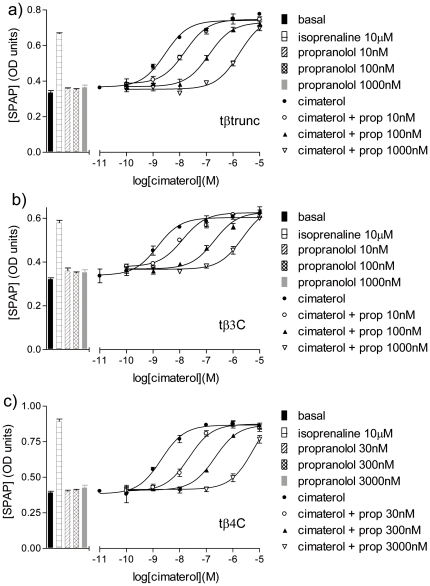
CRE-SPAP production in response to cimaterol in the turkey β-adrenoceptors. CRE-SPAP production in response to cimaterol in a) CHO tβtrunc cells, b) CHO tβ3C cells and c) CHO tβ4C cells in the absence and presence of propranolol. Bars represent basal CRE-SPAP production, that in response to 10 µM isoprenaline and that in response to 10 nM or 30 nM, 100 nM or 300 nM, or 1000 nM or 3000 nM propranolol alone. Data points are mean ± s.e.mean of triplicate determinations. These single experiments are representative of 3 separate experiments in each case. The Schild slopes in these experiments are a)1.10, b)1.15 and c)1.19.

**Figure 6:**
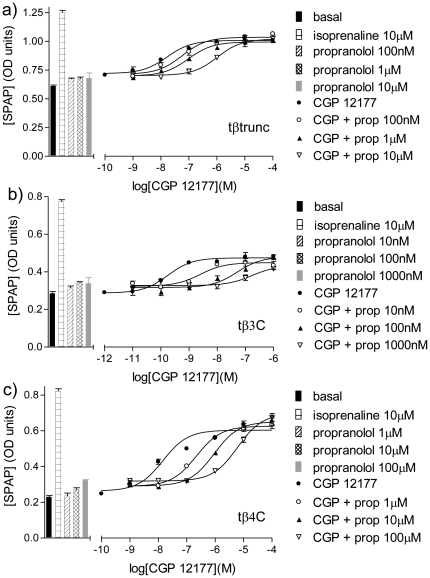
CRE-SPAP production in response to CGP 12177 in the turkey β-adrenoceptors. CRE-SPAP production in response to CGP12177 in a) CHO tβtrunc cells, b) CHO tβ3C cells and c) CHO tβ4C cells in the absence and presence of propranolol. Bars represent basal CRE-SPAP production, that in response to 10 µM isoprenaline and that in response to propranolol (range 10 nM to 100 µM) alone. Data points are mean ± s.e.mean of triplicate determinations. These single experiments are representative of 3 separate experiments in each case.

Isoprenaline stimulated CRE-SPAP gene transcription responses that were 2.34±0.10 (n = 10), 2.30±0.09 (n = 19) and 2.47±0.10 (n = 22) fold over basal for tβtrunc, tβ3C and tβ4C-receptors respectively. The agonist activity of 10 other β-adrenoceptor agonists and CGP12177 was also studied. These agonist responses were then inhibited by the non-selective β-antagonist propranolol, the human β1-selective antagonist CGP20712A and the human β2-selective antagonist ICI118551 (Figure 5 Tables 2–4). At the tβ3C-receptor there is a range of antagonist K_D_ values seen for all three antagonists (e.g. for propranolol, the K_D_ values range from −9.01 to−8.78 when the non-catecholamine agonists are present and −8.45 to −8.13 for when the catecholamines are the competing agonist). This is very similar to that previously reported from the human β2-adrenoceptor in this assay [29]. The K_D_ value for propranolol when CGP12177 is that agonist it −8.95, very similar to that for when the other the non-catecholamine agonists are present. The same pattern is true for CGP20712A and ICI118551. At the tβtrunc and tβ4C-receptors, a similar range in antagonist K_D_ values is seen for when the non-catecholamine and catecholamine agonists are present (again the K_D_ values are lower in the presence of the catecholamines, similar to that in [9], [14]). However, at the tβtrunc and tβ4C-receptors, the concentrations of antagonist required to inhibit the CGP12177 agonist response were significantly higher than the concentrations required to inhibit any the other agonist responses (Figure 6 Tables 2–4).

**Table 2:** Agonist and antagonist characteristics at the truncated turkey β-adrenoceptor.

tβtrunc	Log EC_50_	% isop max	n	Log K_D_ propranolol	n	Log K_D_ CGP20712A		Log K_D_ ICI118551	n	Log K_D_ CGP12177	n
fenoterol	−8.17±0.02	119.4±5.2	3	−8.84±0.05	9	−8.20±0.00	4	−7.43±0.05	5	−9.66±0.09	9
formoterol	−8.96±0.04	115.2±5.1	5	−8.77±0.03	10	−8.20±0.10	5	−7.44±0.09	6	−9.65±0.07	8
BRL37344	−6.45±0.05	107.9±4.2	8	−8.72±0.05	6	−8.04±0.07	75	−7.25±0.08	8	−9.44±0.11	11
cimaterol	−8.63±0.05	122.2±4.6	7	−8.70±0.05	9	−8.24±0.09	5	−7.49±0.04	6	−9.54±0.07	14
clenbuterol	−7.66±0.05	102.1±3.5	12	−8.52±0.05	16	−7.82±0.09	13	−7.32±0.10	12	−9.40±0.07	15
salmeterol	−6.86±0.05	112.2±2.9	13	−8.42±0.08	10	−7.71±0.08	11	−7.15±0.10	9	−9.32±0.10	9
salbutamol	−6.33±0.07	104.8±3.5	10	−8.41±0.10	10	−7.93±0.09	6	−7.19±0.08	6	−9.34±0.11	10
adrenaline	−7.13±0.08	117.2±3.1	8	−8.38±0.12	13	−7.35±0.12	9	−6.82±0.05	7	−9.19±0.06	8
noradrenaline	−7.74±0.04	113.0±4.2	12	−8.35±0.07	16	−7.51±0.08	11	−6.98±0.07	13	−9.05±0.10	11
isoprenaline	−8.11±0.12	100	10	−8.21±0.10	18	−7.25±0.13	8	−6.72±0.12	10	−9.19±0.11	12
dobutamine	−6.53±0.06	123.8±5.1	3	−8.19±0.11	9	−7.53±0.09	5	−6.80±0.09	4	−8.94±0.08	7
CGP12177	−8.38±0.08	53.1±3.6	20	−7.23±0.12	18	−6.91±0.15	12	−6.36±0.17	11		

Log EC_50_ values and % isoprenaline maximal responses obtained from CRE-SPAP production in CHO tβtrunc cells. Log K_D_ values for propranolol, CGP20712, ICI118551 and CGP12177 as antagonists of the agonist responses are also given. Values are mean ± s.e.m. of n separate determinations.

**Table 3 pone-0015487-t003:** Agonist and antagonist characteristics at the turkey β3C-adrenoceptor.

tβ3C	Log EC_50_	% isop max	n	Log K_D_ propranolol	n	Log K_D_ CGP20712A		Log K_D_ ICI118551	n	Log K_D_ CGP12177	n
fenoterol	−9.08±0.08	105.9±3.0	16	−9.01±0.04	4	−5.61±0.11	6	−9.34±0.07	4	−9.94±0.08	9
formoterol	−10.35±0.05	103.6±3.0	19	−8.78±0.05	14	−5.44±0.10	6	−9.29±0.06	5	−9.71±0.07	10
BRL37344	−7.67±0.04	88.5±4.2	16	−9.01±0.12	5	−5.51±0.12	7	−9.31±0.07	4	−9.73±0.11	7
cimaterol	−9.36±0.08	103.1±2.2	17	−8.93±0.08	13	−5.46±0.06	5	−9.40±0.06	4	−9.58±0.07	11
clenbuterol	−9.28±0.06	97.8±4.4	17	−8.95±0.06	13	−5.75±0.06	4	−9.47±0.05	4	−9.78±0.07	10
salmeterol	−10.43±0.06	96.0±5.7	18	−8.90±0.09	5	−5.20±0.12	8	−9.21±0.08	5	−9.76±0.11	7
salbutamol	−8.45±0.06	101.8±3.1	16	−8.90±0.07	4	−5.49±0.10	7	−9.31±0.09	4	−9.71±0.11	6
adrenaline	−7.20±0.09	105.9±2.7	17	−8.24±0.07	11	−5.34±0.09	6	−8.79±0.08	7	−9.37±0.08	10
noradrenaline	−6.38±0.07	101.8±3.0	20	−8.45±0.10	13	−5.28±0.11	5	−8.86±0.11	6	−9.18±0.10	9
isoprenaline	−7.57±0.09	100	19	−8.13±0.09	13	−5.43±0.10	4	−8.60±0.11	8	−9.11±0.11	11
dobutamine	−6.37±0.06	100.8±3.7	15	−8.45±0.09	4	−5.13±0.09	5	−8.94±0.09	4	−9.00±0.09	6
CGP12177	−9.66±0.06	39.9±3.2	19	−8.95±0.08	11	−5.57±0.15	8	−9.50±0.08	10		

Log EC_50_ values and % isoprenaline maximal responses obtained from CRE-SPAP production in CHO tβ3C cells. Log K_D_ values for propranolol, CGP20712, ICI118551 and CGP12177 as antagonists of the agonist responses are also given. Values are mean ± s.e.m. of n separate determinations.

**Table 4:** Agonist and antagonist characteristics at the turkey β4C-adrenoceptor.

tβ4C	Log EC_50_	% isop max	n	Log K_D_ propranolol	n	Log K_D_ CGP20712A		Log K_D_ ICI118551	n	Log K_D_ CGP12177	n
fenoterol	−8.07±0.05	99.9±3.0	20	−8.58±0.07	4	−6.01±0.06	6	−7.33±0.08	4	−9.10±0.11	10
formoterol	−9.78±0.06	96.3±4.5	21	−8.55±0.08	14	−5.90±0.03	6	−7.21±0.10	4	−9.04±0.06	8
BRL37344	−7.15±0.07	97.1±3.0	16	−8.54±0.05	4	−6.04±0.07	6	−7.33±0.08	4	−9.07±0.07	6
cimaterol	−8.70±0.05	98.2±2.3	18	−8.56±0.07	14	−6.00±0.09	5	−7.25±0.02	5	−9.02±0.09	12
clenbuterol	−7.39±0.06	97.2±1.9	21	−8.32±0.07	14	−5.97±0.05	6	−7.19±0.11	7	−8.84±0.09	13
salmeterol	−8.40±0.07	102.5±2.8	18	−8.40±0.11	5	−5.93±0.07	6	−7.27±0.11	5	−8.97±0.05	8
salbutamol	−7.37±0.05	99.9±2.3	20	−8.46±0.05	4	−5.94±0.03	6	−7.38±0.01	3	−9.13±0.08	9
adrenaline	−6.72±0.05	99.6±2.7	18	−7.83±0.09	14	−5.67±0.10	6	−6.83±0.09	7	−8.85±0.10	6
noradrenaline	−7.09±0.09	102.3±2.5	19	−7.59±0.13	13	−5.78±0.08	4	−6.82±0.09	3	−8.58±0.10	7
isoprenaline	−7.88±0.12	100	22	−7.87±0.13	13	−5.74±0.12	7	−6.72±0.12	5	−8.85±0.10	6
dobutamine	−6.80±0.09	110.4±2.4	18	−7.92±0.11	4	−5.63±0.08	4	−6.56±0.08	12	−8.58±0.09	9
CGP12177	−7.83±0.05	53.4±2.2	24	−6.47±0.09	15	−5.30±0.11	10	−6.20±0.06	12		

Log EC_50_ values and % isoprenaline maximal responses obtained from CRE-SPAP production in CHO tβ4C cells. Log K_D_ values for propranolol, CGP20712, ICI118551 and CGP12177 as antagonists of the agonist responses are also given. Values are mean ± s.e.m. of n separate determinations.

**Figure 7:**
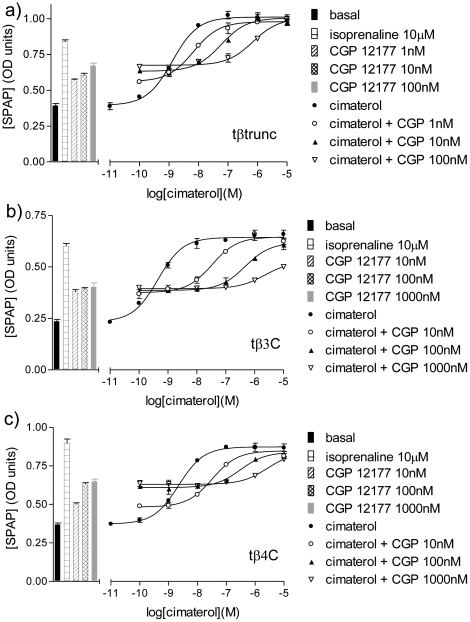
CRE-SPAP production in response to cimaterol in the turkey β-adrenoceptors. CRE-SPAP production in response to cimaterol in a) CHO tβtrunc cells, b) CHO tβ3C cells and c) CHO tβ4C cells in the absence and presence of CGP12177. Bars represent basal CRE-SPAP production, that in response to 10 µM isoprenaline and that in response to 1 nM, 10 nM, 100 nM or 1000 nM CGP12177 alone. Data points are mean ± s.e.mean of triplicate determinations. These single experiments are representative of a) 5, b) 4 and c) 5 separate experiments.

The antagonistic ability of CGP12177 itself was then investigated and was found to be a high affinity partial agonist at all three turkey β-adrenoceptors (Figure 7, Tables 2–4).

Looking carefully at the CGP12177 concentration response curve, this might not be best described by a one-site sigmoidal concentration response curve in all cases (Figure 6). This was therefore investigated further in an assay with a much larger basal to maximum window (see below) where these smaller details are more easily seen.

### ^3^H-cAMP accumulation

S-cyanopindolol was the ligand with which the tβtrunc-receptor X-ray structure was originally solved [23]. It stimulates a two-component concentration response curve at the human β1 and β3-adrenoceptors, but only a one-component sigmoidal concentration response at the human β2-adrenoceptor [30]. The agonist action of this compound was therefore investigated at the three turkey β-adrenoceptors. The ^3^H-cAMP assay was chosen for this as it has a much bigger window (basal to maximum) in which to examine small responses in detail.

**Figure 8:**
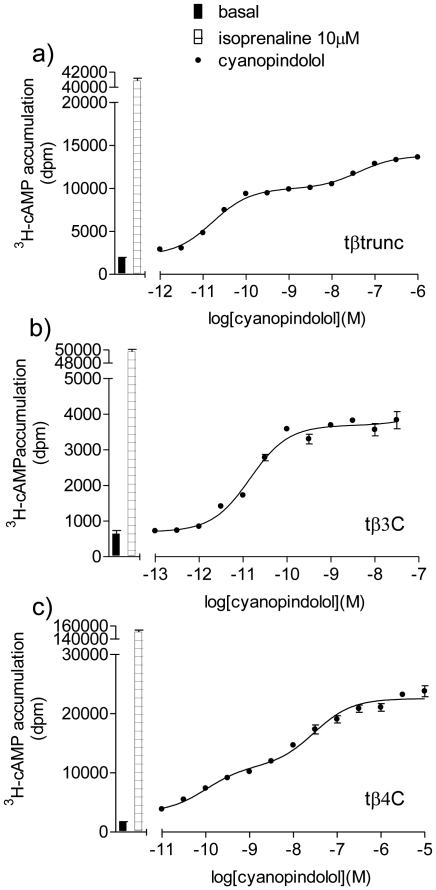
^3^H-cAMP accumulation in response to cyanopindolol in the turkey β-adrenoceptors. ^3^H-cAMP accumulation in response to cyanopindolol in a) CHO tβtrunc cells, b) CHO tβ3C cells and c) CHO tβ4C cells. Bars represent basal ^3^H-cAMP accumulation and that in response to 10 µM isoprenaline. Data points are mean ± s.e.mean of triplicate determinations. These single experiments are representative of a) 4, b) 5 and c) 5 separate experiments.

S-cyanopindolol was found to be a partial agonist at all three turkey β-adrenoceptors and stimulated an agonist response that was best described by a two component response at both the tβtrunc and tβ4C-receptors, but a one-component response at the tβ3C-receptor (Figure 8, Table 5).

**Table 5:** Log EC_50_ values and % isoprenaline maximal responses obtained from ^3^H-cAMP accumulation in the three turkey β-adrenoceptors.

receptor	ligand	Log EC_50_1	Log EC_50_2	% site 1	% isop max	n
tβtrunc	S-Cyanopindolol	−10.84±0.07	−7.41±0.04	65.9±1.5%	36.6±3.4	4
	CGP12177	−9.46±0.11	−7.40±0.10	59.4±2.2%	42.7±2.6	4
tβ3C	S-Cyanopindolol	−10.60±0.13		100%	4.6±0.7	5
	CGP12177	−9.38±0.16		100%	5.3±0.4	5
tβ4C	S-Cyanopindolol	−9.81±0.14	−7.20±0.20	44.4±2.0%	16.4±2.7	5
	CGP12177	−8.73±0.12	−6.58±0.15	41.3±7.2%	17.4±0.7	4

Log EC_50_ values and % isoprenaline maximal responses obtained from ^3^H-cAMP accumulation in CHO tβtrunc cells, CHO tβ3C cells and CHO tβ4C cells. Where responses were best described by a two-component response, Log EC_50_1, log EC_50_2 values, % of response that was occurring via site 1 and % isoprenaline of the overall response are given. Values are mean ± s.e.m. of n separate determinations.

**Figure 9:**
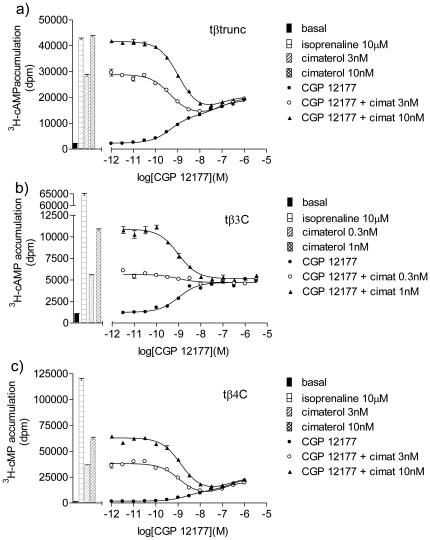
^3^H-cAMP accumulation in response to cimaterol and CGP 12177 in the turkey β-adrenoceptors. ^3^H-cAMP accumulation in response to CGP12177 in a) CHO tβtrunc cells, b) CHO tβ3C cells and c) CHO tβ4C cells in the absence and presence of a fixed concentration of cimaterol. Bars represent basal ^3^H-cAMP accumulation, that in response to 10 µM isoprenaline and that in response to 0.3 nM, 1 nM, 3 nM or 10 nM cimaterol alone. Data points are mean ± s.e.mean of triplicate determinations. These single experiments are representative of a) 4, b) 5 and c) 4 separate experiments.

In this assay with a bigger response window, CGP12177 stimulated an agonist response that was also best described by a two-component response for both the tβtrunc and tβ4C-receptors (Figure 9, Table 5). This is in contrast to the findings at the human β1 and β3-adrenoceptors (where even in assays with a large experimental window, the CGP 12177 response was best described by a one-component concentration response curve). For the tβ3C-receptor, the CGP12177 agonist response was best described by a one-component sigmoidal response curve, similar to that at the human β2-adrenoceptor. In addition, at the tβ3C-receptor, CGP12177 behaved as a typical partial agonist, inhibiting cimaterol at similar concentrations that cause a stimulatory effect, just as would be expected when both ligands (CGP 12177 and cimaterol) are competing at one single site (Figure 9b). At the tβtrunc and tβ4C-receptors, CGP12177 was initially (i.e. at low concentrations) able to inhibit the response to cimaterol at similar concentrations to that required when the first component of stimulation by CGP 12177 is seen. This would be consistent with cimaterol and CGP 12177 competing at the same (“catecholamine”) site of the receptor. However, as the CGP 12177 concentration increases, the overall stimulation by CGP 12177 increases as the second component of the response is seen. This cannot be occurring at the same site as the cimaterol response and first component of CGP 12177 response as further addition of CGP 12177 would then make no difference in overall stimulation (aka tβ3C where CGP 12177 acts via one-site only). This second component stimulation, to the right of the cimaterol inhibition, must therefore be occurring at a different agonist site on the receptors.

### Parent CHO cells

There was no specific binding of ^3^H-CGP12177 to the parent CHO cells (i.e. CHO cells without a transfected receptor). In the ^3^H-cAMP accumulation assay, the parent CHO cells responded to forskolin (21.5±2.2 fold over basal, n = 10) but there was no response to the other ligands.

## Discussion

Ligand binding has long been used to determining which receptors are present in a given tissue [31]. A ligand binds to a certain receptor with a given affinity and this affinity will remain similar whichever tissue the receptor is expressed in. If a range of ligands are examined, a pattern, similar to a fingerprint, is obtained for a given receptor. Figure 3 shows the affinity of 48 ligands at the turkey receptors. Although the tβtrunc and tβ4C-receptors have distinct amino acid sequences, they are the most pharmacologically similar of the turkey β-adrenoceptors. Thus the turkey β-adrenoceptor [15] is distinct (in sequence and/or binding) from either the turkey β3C or β4C-adrenoceptors [19] as indeed the tβ3C and tβ4C-receptors are from each other. The turkey therefore has three distinct β-adrenoceptors.

Figure 4 shows a comparison of the same 48 ligands with the affinity for the human β1, β2 and β3-adrenoceptors. Here, it can be seen that the turkey β3C-receptor has a similar affinity pattern to that of the human β2-adrenoceptor. The affinity of ligands for the tβ-trunc and tβ4C-receptor do not correlate as well with any of the human β-adrenoceptors and thus the tβtrunc and tβ4C are distinct β-adrenoceptors. Figure 2 demonstrates this with two individual ligands (CGP20712A and ICI118551) which are highly selective for the human β1 and β2-adrenoceptors respectively. Interestingly this species effect is seen elsewhere - ICI118551, whilst having high affinity for the human β2-adrenoceptor has lower affinity for the porcine β2-adrenoceptor [32]–[34].

The β1-adrenoceptor [2]–[13] (and human β3-adrenoceptor [14]) have been shown to exist in two different conformations. Agonist responses occurring at the high affinity catecholamine site are blocked by traditional β-blockers whilst agonist responses occurring via the secondary low affinity site are relatively resistant to antagonism. The presence of both of these sites has been reported in transfected cell systems (e.g. [1] [6] [35], including work from this laboratory [9] [11], [14]), isolated cells (e.g. rat cardiomyocytes, [36]), tissues (e.g. mouse brown fat [35] ferret myocardium [7], rat artery [37]) and in whole animals (including mice [38]–[39] and humans [40]). Several β-adrenoceptors have been shown to activate different downstream signalling (“dual efficacy ligands” e.g. human β2 [41]–[42] and mouse β3 [43] but this is different from the high affinity/low affinity states of the β1 and β3-adrenoceptors outlined above.

There are four lines of evidence for the existence of a secondary low affinity or CGP12177 site on β-adrenoceptors from functional assays. Firstly, the EC_50_ for the CGP12177 stimulatory response is not the same as the CGP12177 K_D_ value for antagonism of other agonists [6], [11]. If only one site is present on a receptor, the EC_50_ of a partial agonist (concentration at which half the response is stimulated) is the same as the K_D_ value (concentration at which half of the receptors are occupied). A full, more efficacious agonist would only need to occupy only a few receptors in order to elicit a response, and thus the EC_50_ value will be lower than the K_D_ value. CGP12177 stimulated a partial agonist response at all three turkey β-adrenoceptors. For the tβ3C-receptors, the EC_50_ value for CGP12177 (−9.66) is within the range of K_D_ values obtained from antagonism of the more efficacious agonists (−9.11 to −9.94) suggesting that CGP12177 is a typical partial agonist causing its stimulation and antagonism by occupying the same conformation of the receptor (which is also the same conformation occupied by the other agonists). This is similar to that reported for the human β2-adrenoceptor [24]. However for the tβtrunc and tβ4C-receptors, CGP12177 has an EC_50_ value that was significantly greater than the K_D_ value (obtained in the same assay), similar to that reported for human and rat β1-adrenoceptors [6] [11]. This would therefore suggest that for the tβtrunc and tβ4C-receptors, the CGP12177 stimulation is occurring at a different place (requiring more ligand and so higher affinity) than when CGP12177 is inhibiting the other agonists.

The second piece of pharmacological evidence for more than one conformation is the concentrations of antagonists required to inhibit the CGP12177-stimulatory response. If the CGP12177 partial agonist response was occurring at the same conformation as the other agonist responses, they would require the same concentrations of other antagonist to inhibit it (and thus the K_D_ value of other antagonists would be the same regardless of which agonist was used). For the tβ3C-receptor, the K_D_ values of the antagonists (propranolol, CGP20712A and ICI118551) are the same for when CGP12177 is the agonist as to when the other 11 agonists are used (Table 3, also as for the human β2-adrenoceptor [24]). However, the concentrations of propranolol, CGP20712A and ICI118551 required to inhibit the CGP12177 response at the tβtrunc and tβ4C-receptors was significantly greater (leading to lower K_D_ values) than those required to inhibit the other 11 agonist responses (Tables 2 and 4). This is similar to observations at the β1-adrenoceptor in rodents and mammals [6]–[7] [9] [11]. This would suggest that the CGP12177 agonist response was occurring at a different pharmacological site to the other agonist responses. There was no response to any ligands, including CGP12177 in the parent CHO CRE-SPAP cells and thus the CGP12177 agonist response was not occurring at a different receptor natively expressed in the cells.

The third line of evidence comes from agonist response curves that contain more than one component. Several ligands have been previously shown to stimulate a two-component concentration response curve at the β1-adrenoceptor (e.g. alprenolol, bucindolol, carazolol, S-cyanopindolol, oxprenolol, pindolol, S-pindolol and SDZ21009 [10]–[11] [30]. Several others have been shown to stimulate a two component response at the human β3-adrenoceptor [14] [30] but to date none have been shown to stimulate a similar two-component response at the human β2-adrenoceptor. S-cyanopindolol stimulated an agonist response that was best described by a two-component response at both the tβtrunc and tβ4C-receptors. The S-cyanopindolol response at the tβ3C-receptor is best described by a one-component response. This therefore suggests that S-cyanopindolol is able to stimulate a response at two agonist conformations of the tβtrunc and tβ4C-receptors. When the agonist response to CGP12177 was examined in more detail in the cAMP assay (with a larger window to examine the response) it can be seen that it too stimulates a response best described by a two-component response at both the tβtrunc and tβ4C-receptors.

Finally, if the tβtrunc and tβ4C-receptors do indeed exist in two agonist conformations and CGP12177 is able to bind to both conformations (the first “catecholamine” conformation with high affinity and the secondary conformation with lower affinity), then it should be possible to demonstrate inhibition of a more efficacious catecholamine-site agonist at low concentrations of CGP12177, separately from the second site stimulatory response at higher concentrations of CGP12177 [1] [6] [9] [11] [14]. This is exactly what is seen for both the tβtrunc and tβ4C-receptors (Figure 9a and c). This pattern is not seen for the tβ3C-receptor, where inhibition of the agonist by CGP12177 occurred at a similar concentration as the stimulatory effect of CGP12177, suggesting that the two are indeed occurring at the same site (Figure 9b).

Thus the tβtrunc and tβ4C-receptors appear to exist in more than one agonist conformation, similar to that seen at the human and rat β1-adrenoceptor and the human β3-adrenoceptor [1] [6] [9] [11] [14]. The tβ3C-receptor appears to exist in only one agonist conformation, similar to that for the human β2-adrenoceptor [24]. Overall there are therefore several different β-adrenoceptors: human β1, human β3, tβtrunc and tβ4C-adrenoceptors that are distinct β-adrenoceptors, as defined by their binding characteristics and/or amino acid sequence, but that all exist in at least two agonist conformations. The human β2 and tβ3C-receptors have very similar binding characteristics and appear to exist in only one agonist conformation. Comparison of the amino-acid sequences of these receptors, and others already known to exist in two conformations e.g. rat and ferret β1-adrenoceptors [6]–[7], should therefore help in determining which residues are required for the existence of the second conformation.

In conclusion, there are three distinct turkey β-adrenoceptors. Of these the greatest similarity of any of the turkey β-adrenoceptors to human β-adrenoceptors is between the turkey β3C-receptor and the human β2-adrenoceptor. There are pharmacologically distinct differences between the binding of ligands for the tβtrunc and tβ4C and the human β-adrenoceptors. The tβtrunc and tβ4C-adrenoceptors appear to exist in at least two different agonist conformations in a similar manner to that seen at both the human and rat β1-adrenoceptor and human β3-adrenoceptors. The tβ3C-receptor, similar to the human β2-adrenoceptor, does not, at least so far, appear to exist in more than one agonist conformation. Comparison of the amino-acid sequence for the turkey and human adrenoceptors may therefore shed more light on the residues involved in the existence of the secondary β-adrenoceptor conformation.

## Supporting Information

Figure S1 **Correlation plot of the log K_D_ values for the human β-adrenoceptors compared with each other.**
Correlation plot of the log K_D_ values for the ligands in Table 1 for a) human β1 vs human β2-adrenoceptor, b) β1 vs human β3-adrenoceptor and c) β2 vs human β3-adrenoceptor.
Ligands are labelled as full agonists if the stimulated more than 90% of the response at the human β1-adrenoceptor, as partial agonists if they stimulated 5–90% of the full response and as antagonists if they stimulated less than 5% of a full agonist response. Data are from [25] [30].
(TIF)
