# Supplementary figures and images for: A Full Pharmacological Analysis of the Three Turkey β-Adrenoceptors and Comparison with the Human β-Adrenoceptors
Source: PLoS One. 2010 Nov 30;5(11):e15487. doi: 10.1371/journal.pone.0015487 (PMC2994877; doi:10.1371/journal.pone.0015487)

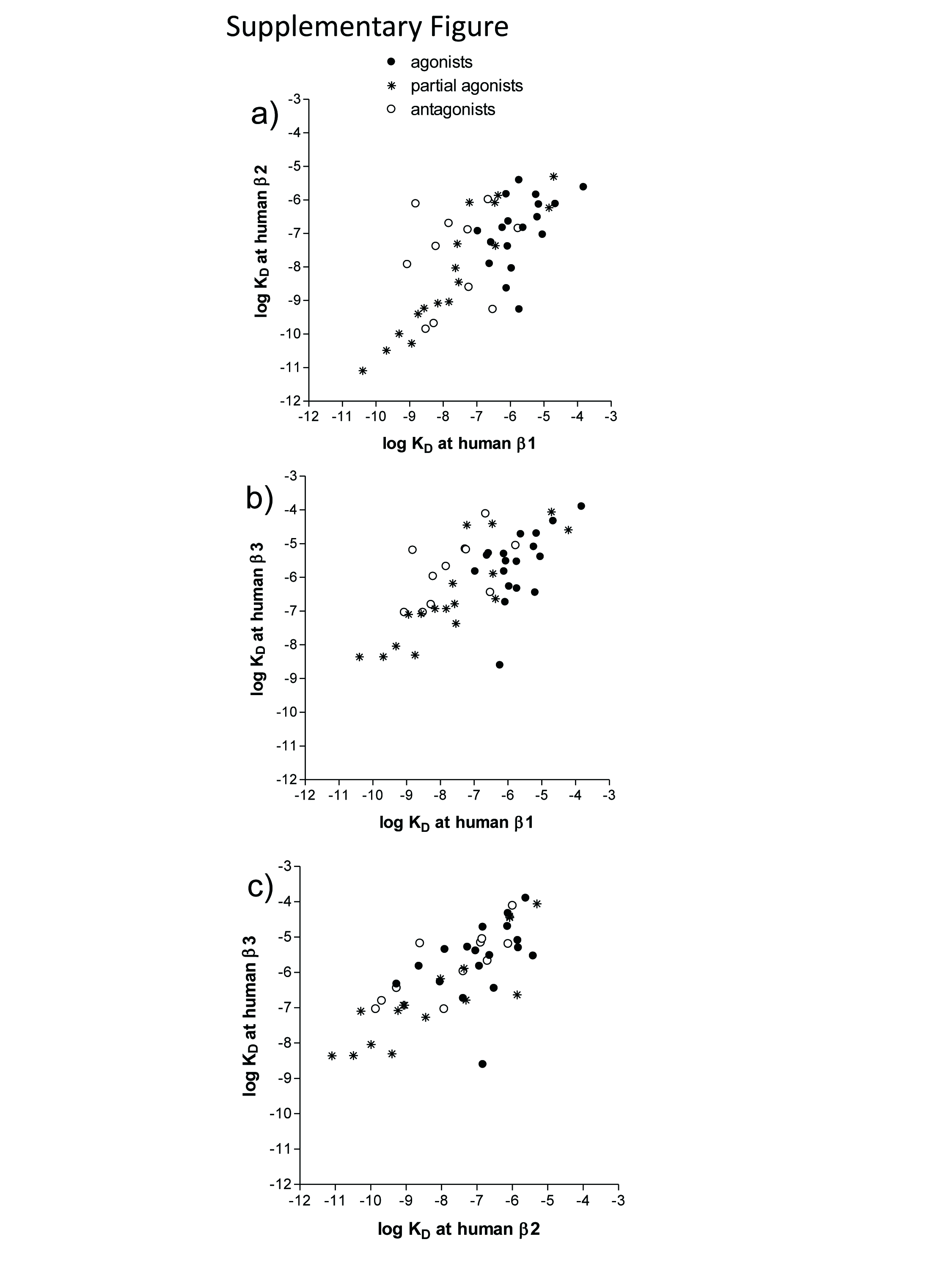

Supplement: Figure S1 — Correlation plot of the log KD values for the human β-adrenoceptors compared with each other. Correlation plot of the log KD values for the ligands in Table 1 for a) human β1 vs human β2-adrenoceptor, b) β1 vs human β3-adrenoceptor and c) β2 vs human β3-adrenoceptor. Ligands are labelled as full agonists if the stimulated more than 90% of the response at the human β1-adrenoceptor, as partial agonists if they stimulated 5–90% of the full response and as antagonists if they stimulated less than 5% of a full agonist response. Data are from [25] [30]. (TIF) [file pone.0015487.s001.tif]
